# Experiences of breast milk donors in Sweden: balancing the motivation to do something good with overcoming the challenges it entails

**DOI:** 10.1186/s13006-024-00668-3

**Published:** 2024-08-31

**Authors:** Ylva Thernström Blomqvist, Emma Olsson

**Affiliations:** 1 Neonatal Intensive Care Unit, University Children’s Hospital, Uppsala, Sweden; 2 Department of Women’s and Children’s Health, Uppsala University, Uppsala, 751 85 Sweden; 3 Department of Pediatrics, Faculty of Medicine and Health, Örebro University, Örebro, Sweden; 4 Faculty of Medicine and Health, School of Health Sciences, Örebro University, Örebro, Sweden

**Keywords:** Breast milk donation, Experiences, Human breast milk, Mother’s milk, Neonatal care

## Abstract

**Background:**

Infants requiring neonatal care often face initial breastfeeding challenges, leading them to receive expressed breast milk from their mother or donor milk. While emphasizing the mother’s own milk as the gold standard for infant nutrition, the utilization of donor milk stands as the preferred alternative over infant formula due to its numerous benefits. To facilitate the provision of donor milk to preterm and ill infants in neonatal units, the active participation of women willing to contribute their breast milk is crucial. This study aims to enhance the understanding of women’s experiences in the donation process, thereby contributing to efforts aiming at alleviating the shortage of donated breast milk by improve the care and support for breast milk donors.

**Methods:**

This descriptive qualitative study took an inductive approach based on individual semi-structured interviews conducted during 2021 with 15 breast milk donors in Sweden. The data were analysed with thematic analysis.

**Results:**

Two themes were identified in the analysis: motivation to donate and challenges to overcome. Many of the women struggled to overcome the apparent challenges of not only starting the process of donating breast milk but also maintaining it. Despite the strain, they were motivated to donate their breast milk and seeking information by themselves to do something important for someone else. Only a few of the women talked about the financial benefits of donating breast milk; donating seemed to be mostly based on altruistic reasons.

**Conclusions:**

Despite the challenges posed by COVID-19 restrictions, time consumption, and the hard work of sterilizing pump utensils, women continued to donate their milk driven by altruism. To enhance donor support and increase milk donation, several improvements are suggested: providing comprehensive information and resources, simplifying the donation process, offering flexible scheduling, and recognizing donors’ contributions.

**Supplementary Information:**

The online version contains supplementary material available at 10.1186/s13006-024-00668-3.

## Background

Breast milk is the ideal nutrition for all newborn infants [1, 2]. The optimal choice for feeding infants in a neonatal intensive unit (NICU) is the mother’s own milk (MOM) as the primary option. However, it is essential to underscore that in cases where breastfeeding or MOM is not feasible, donated breast milk (DM) stands as the foremost alternative, prioritized as the first-choice supplement for providing essential nourishment to these infants in need of care [2–4]. Infants fed infant formula have higher rates of various diseases such as sepsis, respiratory infections, and urinary tract infections than infants fed human milk only [5]. In addition, for infants in the NICU, full enteral feeding is achieved earlier if breast milk is given, which can reduce the time spent on parenteral nutrition [6]. Breast milk also contains antibodies which provide significant protection against any common childhood diseases that the woman herself has already had [7]. Further, breast milk provides protection against diseases such as NEC (necrotizing enterocolitis) [8], which is one of the leading causes of neonatal mortality [9]. Moreover, breast milk improves the cognitive development of infants born prematurely [10]. Infants in need of neonatal intensive care often have difficulties breastfeeding directly, and may therefore initially receive MOM or DM through a feeding cup or tube [11]. While DM presents certain disadvantages when compared to the MOM, its considerable advantages over infant formula make it a preferable alternative in situations where breastfeeding or MOM is not achievable. In order to provide DM to preterm and sick infants at the NICU, women who are willing to donate their breast milk are essential. Donating breast milk can be a demanding process which requires emotional and practical support from health care professionals in order to maintain the donors’ motivation [12–14]. Milk donors who receive support from family members and friends have been found to donate greater amounts of milk [13]. However, many breast milk donors struggle to find the necessary information and find the breast milk donation process strenuous. The donation of milk also diverts time from their own infant [12]. Doshmangir, Naghshi and Khabiri reported that there can be cultural and religious barriers to donating breast milk [15].On the other hand, some motivating aspects for milk donation are reported in the literature such as altruism, excess milk, and a willingness to help [15]. There are also women who feel proud and strong and experience the breast milk donation as positive and valuable [14].

In Sweden, there is a culture and a norm of providing breast milk to newborn infants [16], but there is still a need of DM for infants at NICUs. Despite a good collaboration between the respective milk banks in Sweden, with opportunities to buy DM from each other, there are still periodical shortages of DM. The reason for this is unknown and the lack of DM can put the preterm and sick newborn infants at risk of receiving sub optimal nourishment. The findings from this study will contribute to the international literature by providing insights into the donation process from a Swedish context, which could be applicable and beneficial to other settings facing similar challenges. This study aims to enhance the understanding of women’s experiences in the donation process, thereby contributing to efforts aiming at alleviating the shortage of donated breast milk by improve the care and support for breast milk donors.

## Methods

### Aim

The aim was to enhance the understanding of women’s experiences in the donation process, thereby contributing to efforts aiming at alleviating the shortage of donated breast milk by improve the care and support for breast milk donors.

### Design

This was a descriptive qualitative study with an inductive approach.

### Setting

The Swedish parental leave policy is notably generous, providing parents with a total of 480 days of paid leave per child. Ninety of these days are specifically allocated for each parent [17]. In Sweden 93% of newborns are breastfed one week postpartum, and of this population, 72% are exclusively breastfed. As children reach 6 months of age, 65% continue to breastfeeding to some extent [16]. Notably, a recent Swedish study demonstrated an association between an extended shared parental leave and an increased duration of breastfeeding, highlighting the positive impact of parental leave policies on infant feeding practices [18]. The milk banks in Sweden are locally situated at the respective hospital and can either be organized by staff from the NICU or affiliated to the nutrition department at the hospital. Depending on the unit policy the women can receive economic compensation for the breast milk she donor and might also be supported with collection of the breast milk instead of having to deliver the milk to the milk bank themselves. All presumptive donors undergo mandatory screening with blood tests for HIV-1, HIV-2, HTLV-I, HTLV-II, hepatitis B, and hepatitis C before the donation can begin, and the DM is also bacterial culture test and pasteurised prior to use [19]. The two milk banks in this study have different routines where one is situated at the NICU and one is located centrally at the hospital’s nutrition department, the compensation and service provided by the milk bank also differs. Both milk banks pasteurize (Holder pasteurization) the donated milk before use [19]. One of them provides financial compensation to the donor, offering SEK 150, while the other offers SEK 250 (approximately US$14–24) per litre of donated milk, tax-free.

### Data collection

A purposive selection was used, both in terms of the two milk banks with different prerequisites and also in the selection of women where we strived for including women with different experiences in terms of length of donation, previous experience with donating milk and women who could provide detailed descriptions in order to answer the study’s aim. The two milk banks were selected for convenience reasons as the two researchers worked at these hospitals. The women were identified through the milk bank registers. The data were collected via individual semi-structured interviews in Swedish between February and August 2021. We utilized an interview guide (see additional file 1) developed from the authors’ extensive clinical experience as female registered nurses, each with over 20 years of practice at the NICUs at the hospitals where the milk banks are located. The guide was also informed by questions that remained unanswered in a previous survey study [12]. The interviews were carried out by the two authors as well as by two specialist registered nurses, all women and with considerable experience from working in neonatal intensive care but without any working experience or connection to the respective milk banks. After the interview the women was asked about the demographic information. We reached out to 18 eligible breast milk donors (all donating women at the two milk banks during 2020), of whom 15 consented to participate in the study. They received detailed study information, sent to their home address, and were contacted by phone to arrange interview appointments. Those who agreed to participate returned the signed consent form either by letter or directly to the interviewer. Interviews were conducted based on the donor’s preference: digitally via Zoom, over the phone, or in person at a hospital. Only the participant and the researcher were present during the interview. No repeated interviews were performed nether was any field notes taken. The interviews lasted between 20 and 58 min and were digitally recorded and transcribed verbatim shortly after being conducted. Participants were recruited until no new relevant knowledge was being obtained.

### Analysis

The transcribed interviews were analyzed with thematic analysis according to the framework presented by Braun and Clarke [20]. The analytical process consisted of six phases, the first five of which involved analyzing the data material to identify and describe themes based on the purpose of the study. The sixth and final phase was report writing. In the first phase of the analysis, the material was read several times by both authors separately in order to get acquainted with the data, and initial thoughts were written down. The second phase involved systematically producing codes throughout the data material by identifying words and sentences which were relevant to the aim of the study. These codes characterized features of the data material. In the third phase, the codes were analyzed by grouping them into possible themes, and in the fourth phase these themes were refined and revised. During the fifth phase, the themes were defined, and the main features of each theme were established. Phases 2 to 5 was done by both authors together and the analysis was carried out manually with post-it notes and pens and in the computer program Word.

### Ethics

Approval was obtained from the Swedish Ethics Review Authority (ref: 2019–02447). The study participants received both written and verbal information about the study, and were informed that their participation was voluntary and that they could withdraw their consent at any time. All participants gave their written consent.

## Results

The participants were between 25 and 37 years old, had between one and five children (Table 1) and the distance from their home to their milk bank was between 2 and 50 km. Two of them had been breast milk donors previously.

**Table 1:** Demographic description of the study participants (*n* = 15)

	m (min–max)	*n*
Age	31.7 (25–37)	
Number of children	2.2 (1–5)	
Civil status		
Living together with the other parent		14
Single parent		6
Employed		
Yes		13
Student		
Yes		2
Level of education		
University/college education		13
Senior high school		2
No previous experience of breast milk donation		13

Min = minimum; Max = maximum

**Fig. 1:**
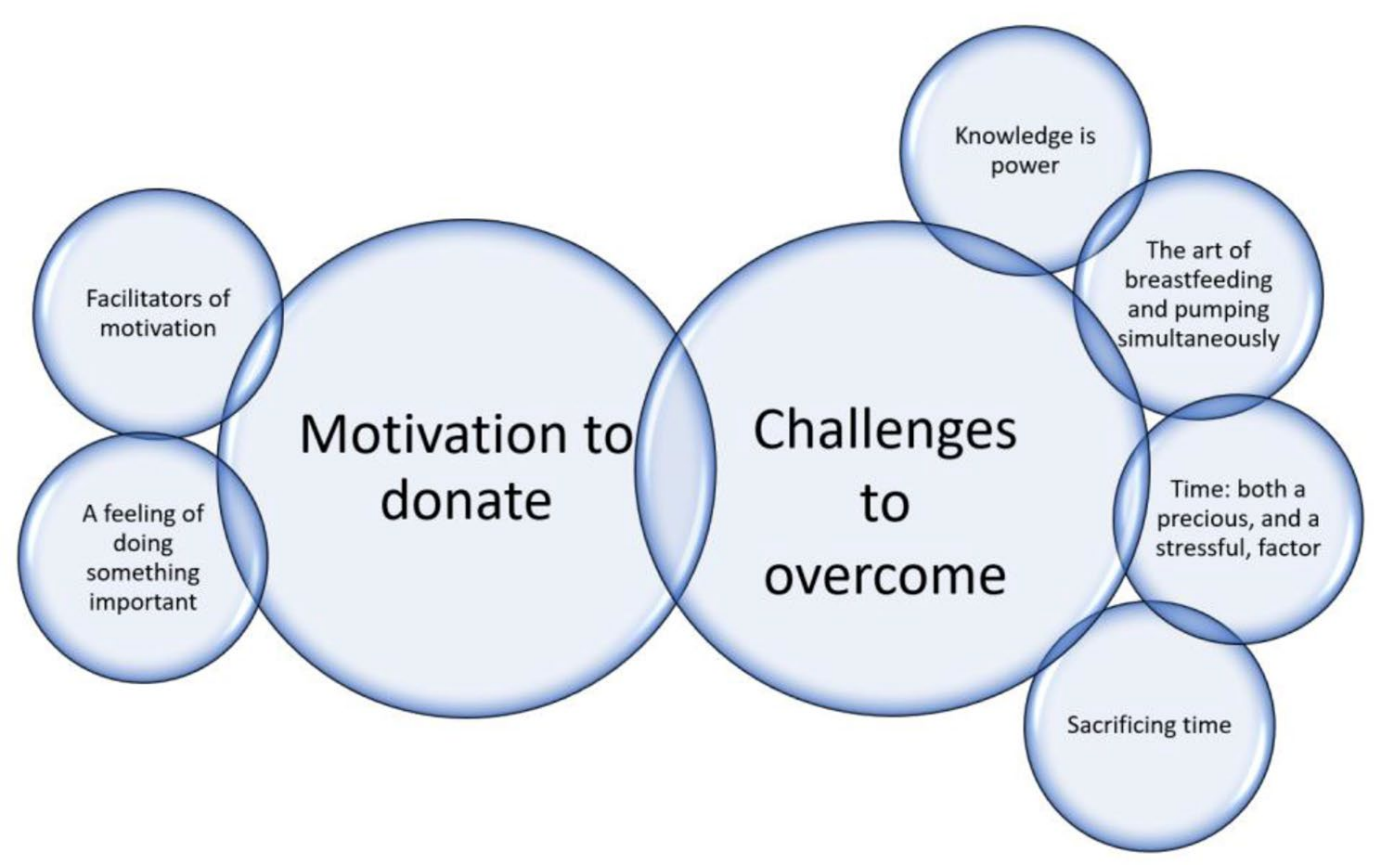
The two themes and six subthemes

The analysis revealed two themes: *motivation to donate* and *challenges to overcome* (Fig. 1).

### Motivation to donate

#### Facilitators of motivation

The staff that the women met in various parts of the health care system were important for their motivation to donate. When they felt seen and taken care of and also had regular contact with the staff at the milk bank, they felt encouraged to start and continue the process of donating. One of the breast milk banks kept in contact with the women during the whole donation process in order to support and encourage them, and the donors greatly appreciated this. Personnel at the breast milk bank were described as pleasant and encouraging by some donors, while others had the opposite experience and experienced a lack of psychological and practical support.

*They contacted me… They contacted me every week and checked the situation and asked if I had any questions.*

Informant Ö4.

A supportive partner was also very important. The donation process was described as a team effort, and several women said that donating would not have been possible without their partner’s support.

My husband had to be part of it because he did half the work, we already knew that he would have to do the dishes and such.

Informant U5.

In addition to the support from staff and partners, there were other important elements that facilitated the process and made the women feel motivated to continue donating; for example, that their breastfeeding worked well and felt good. In addition, there were purely practical facilitators such as being able to borrow a breast pump for free, having the breast milk picked up from their home or the milk bank staff coming out to the donor’s car to pick up the milk so they did not have to look for parking, and transporting both the milk and also take their newborn infant with them to the milk bank.

#### A feeling of doing something important

The incentive to donate was often described as a wish to help someone else, and the fact that it felt good to help others.

The main driving force is of course to help… That I have something that someone else needs and it costs me so little to give it.

Informant Ö1.

One woman who had NICU experiences with her previous children talked about wanting to “give something back” and said that it felt rewarding to nurture other infants. There were also comparisons with donating blood, as two of the women were not eligible to donate blood and instead recognized the breast milk donation as a proxy. Some women had an excess of breast milk that they had already expressed and chose to donate this excess; these were mainly women who had an infant at the NICU. They recognized that they were donating the breast milk that was not needed for their own infant, which felt good. It also felt good to know that they were helping others, and they saw this as a way “to say thank you for the care” their infant had received. A few of the women expressed a feeling of having a lot of spare time, as they were on parental leave with their infant and had the time needed for the donation process. Some were also aware there was a lack of breast milk at the breast milk bank and knew the importance of DM for the babies in the NICU, which further motivated them to donate.

I’ve always wanted to donate blood but couldn’t because of an intestinal disease…. but now I felt that maybe I could donate breast milk anyway.

Informant U6.

### Challenges to overcome

#### Knowledge is power

Many of the women were highly motivated to donate breast milk, but struggled to find information about how to become a breast milk donor.

It felt so complicated, but I was so motivated. I felt that if I hadn’t been so motivated [to donate] I would have just said no….

Informant U7.

Several of them had experienced a lack of knowledge about breast milk donation among staff they met within the health care system. Some said that even at the maternity ward they had not received information about the possibility to donate breast milk, and had not been given any help to get started. A few of the women were informed about the possibility of donating breast milk through flyers or information on screens in the waiting room at the child health care center (a health service that the majority of Swedish children visit regularly during their childhood). However, most of the women struggled to find information about how to begin the process of donating milk. They sought information by themselves from different health care institutions (for example child health care centers) and maternity units, but were mostly met with ignorance. Several steps were often involved, as the women began asking for information at the child health center and then continued on to other medical institutions or searches on the internet in order to find information. One woman who received information about breast milk donation was actually dissuaded from donation by staff at the child health center, who instead encouraged her to focus on her own child’s need for breast milk.

The staff in child health care and maternal health care need more information about this. I think they’re afraid because there are so many women who feel forced to breastfeed, and therefore they don’t dare…. they’re afraid of putting more pressure on new mothers….

Informant Ö1.

Some who did not receive information from health care instead searched for information on social media, such as Facebook. One woman was part of a maternity group on Facebook, and learned there about the possibility of donating breast milk.

Several of the women had experience of the NICU either with the current child or with previous children, or had family members or friends who had been at the NICU and therefore knew the importance of donated breast milk for these patients. One of the women was asked by the staff at the NICU if she would be willing to donate due to her excess of breast milk.

Regarding the information that was available, several of the women thought that the information should have been formulated in a more positive way, more individual, and more based on facts about why DM is so important for infants in need of NICU care.

### The art of breastfeeding and pumping simultaneously

Many of the women breastfed their own babies at the same time as donating breast milk. Getting this together in terms of time and practicality was a challenge for many, and required a lot of patience. All women, even those whose babies were being cared for in the NICU and who did not breastfeed during the donation period, described the expressing and hygienic handling of the breast milk as a time-consuming process. It also took a significant period of time before they found working routines.

The pumping itself didn’t take long, but it was just that you had to clean everything….

Informant U9.

Several women breastfed their infant on one breast at the same time as pumping from the other, which worked well for most of them after a little practice. However, one woman described how her infant was so disturbed by the pump that it did not work. Those who had already donated breast milk before thought it went better this time, as they were better prepared regarding what worked and what felt good, and what did not.

I found it easier to pump if I was breastfeeding at the same time. If I only pumped, I didn’t express nearly as much milk.

Informant U3.

### Time: both a precious, and a stressful, factor

When the study was carried out, there was a time limit for donation in Sweden, and breast milk could only be donated until their infant was three months old. Most of the women stopped donating when the “time ran out”, although several of them said they would have liked to continue for longer. Many of them found this three-month limit to be stressful.

It was because he got too old for me to donate, so I wasn’t allowed to donate anymore, but I would have liked to have continued donating if I had been allowed to.

Informant U10.

One woman stopped donating because she felt she wanted to spend all her time and focus on her own infant. Some of the women stopped donating as their infant no longer needed NICU care and had started breastfeeding, and therefore they no longer needed to pump breast milk to feed their own infant.

Donating also took up valuable family time, and the restrictions during the COVID-19 pandemic added to this. One of the women did not have the time to drop off the milk herself because she was not allowed to bring the infants’ siblings into the hospital where the milk bank was located.

In the end my husband had to drop off the milk, they only had opening hours until 1 or 2 PM, and this was during the pandemic so I wasn’t allowed to bring my older daughter, which made it impossible due to her daycare hours.

Informant U2.

### Sacrificing time

There were many things that were small in themselves but that could add up to make it feel overwhelmingly difficult to donate breast milk. These things were related to, for example, taking care of and breastfeeding a newborn infant, taking care of the infant’s older siblings, pumping breast milk, washing all of the pumping equipment, and taking the milk to the hospital. The process of being a breast milk donor could be both mentally draining and physically strenuous.

Being a new mother could be difficult, with disturbed sleep as well as postpartum physical pain in the back, perineum, and abdomen. Some of the women felt stressed by having to cope with all of this, as well as the pressure to donate enough, since one of the milk banks had a minimum amount required to donate. They wanted to be both good new mothers and good donors. Donation required a balancing act in life, and several women said they managed this thanks to having high self-confidence.

Although these women said that they donated out of free will and because they felt good about doing something important, several also said that they should have received a higher financial compensation than they did. Many would have liked more appreciation and better support and feedback when the donation period was over, for example a thank you card or a text message like the one blood donors in Sweden receive when their blood has been used.

. they could have sent you a text message. It would have been encouraging to know that it [the breast milk] had been used, even if I already suspected that it had.

Informant U9.

## Discussion

This study sought to explore Swedish women’s experiences of donating breast milk. The women spoke openly about their encounters and wanted to share their experiences in order to improve routines and make the process easier for other breast milk donors in the future. Donated breast milk can reduce the risk of a number of morbidities, and could potentially be life saving for preterm infants at the NICU [2, 21]. From the findings of this study, it is therefore worrisome that some women had to struggle to overcome the apparent challenges of both starting and maintaining the process of donating breast milk. Despite the strain, they were motivated to donate their breast milk and seeking information by themselves in order to do something important for someone else.

There are periodic shortages of DM in Swedish NICUs, making it crucial to have enough donors. There is an obvious need to provide more information to women in the target group, and the goal must be for all health care personnel who come into contact with lactating women to be able to at least guide the women towards the closest milk bank. One of the milk banks in the study offered a transport service, where either the DM was collected at the woman’s home or the woman could deposit the DM at a nearby health service which would then forward the DM to the milk bank. This was appreciated by the women as a practical facilitator for a more convenient donation process. Due to the importance of a steady flow of DM into the milk banks and the potential costly consequences of not having it, this transport service might be an effective facilitator for all milk banks. There are economic benefits associated with milk banks and the cost of managing a donor milk bank is lower than the expenses incurred from NICU hospitalizations and its associated risks like NEC [22]. However, practical obstacles in delivering donated milk to these banks, such as distance and parking issues, along with the demands of caring for a newborn, could pose challenges for donors [12]. In Sweden, where mothers of preterm infants usually stay with their infants in the NICU around the clock, the challenge of delivering expressed breastmilk to the NICU is effectively eliminated. Conversely, in other settings, the time required to express breastmilk and transport it from home to the NICU can present a barrier [23].

It was considered important for the staff working in the milk bank to be flexible and to adapt to the individual donor’s needs. The practical aspects of breast milk donation are pivotal for donors, where attention to details is crucial. Donors must comprehend the functionality of the breast pump, determine the appropriate size of the breast funnel, and navigate the practicalities of pumping, especially in conjunction with breastfeeding an infant. Similar to the importance of personalized one-to-one support regarding breastfeeding [24], it is evident that donors also require high-quality, individualized support. Moreover, when it comes to postpartum physical recovery, this aspect could be overlooked by the staff the new mother meets during the first weeks after giving birth. For example, about 50% of first-time mothers still experience back pain 8 weeks postpartum [25].

Only a few of the women talked about the financial benefits of donating breast milk; donating seemed to be mostly based on altruistic reasons, which is in line with studies on women in other cultural contexts [26, 27]. The challenges associated with donor recruitment and retention persist in both breast milk and, for example, blood donation, despite extensive research on the motivations and obstacles related to blood donation [28]. Some individuals find fulfillment and increased happiness in engaging in altruistic behaviors, even those demanding physical sacrifices, such as donating time, blood, or organs [29]. Additionally, a motivation for blood donation includes a sense of reciprocity, where individuals feel compelled to give back as a way of expressing gratitude for personal or significant other’s past assistance [30]. In line with this Olsson et al. [12] found that some women who donate breast milk are concerned with the ethical aspects of giving away something that belongs to their own infant and that he or she may need later. Conversely, the women in the current study expressed a moral obligation to give something back, which might be described as a feeling of righteousness or as a feeling of being in debt; to our knowledge, this has not been shown in other studies. Pimenteira Thomaz et al. [31] reported that a large proportion of women understood the infants’ needs for DM and recognized this as the major incentive for donating breast milk. Wanting to do something for someone else was expressed by the women in the present study. This is in line with the results reported by Gribble [32], where women described this along with other altruistic reasons to donate breast milk. A few women in the present study also described personal benefits of pumping breast milk to donate, due to an excess of breast milk bringing issues both with leakage of milk and with breastfeeding. The lack of breast milk donors is not a Swedish phenomenon, but is also recognized in other countries and cultures; for example, in China [27]. However, one practice that is less common in Sweden is the sharing of breast milk outside of milk banks, where mothers use social media to find donors willing to share/sell their milk [33, 34]. It is possible that this is more common in cultures and countries where the concept of milk banks is not that widespread. However, uncontrolled milk sharing can bring a risk of contamination of the milk due to lack of pasteurization and testing of the milk [34]. In line with the present study, a recent review identified significant barriers for potential breast milk donors, including a lack of information, insufficient practical and psychological support. The review also highlighted that limited knowledge about milk banking among healthcare staff is recognized as a barrier to milk donation [35]. So, determining whether the shortage of donated breast milk stems from a lack of donors or is influenced by a lack of knowledge among healthcare staff poses a challenge. A lack of knowledge among healthcare professionals about the possibility of donating breast milk and how it is performed in practice most likely leads to that women who want to donate receiving less information and support. Therefore, it seems reasonable that staff who meet expectant and new parents need to gain knowledge about the need for donated breast milk and thereby the need for donors.

Today, some mothers feel pressure to breastfeed their infants, and a few of the women in the present study thought this was part of the reason why health care staff seemed reluctant to encourage them to donate breast milk. People working in health care might be afraid to push women towards anything that could potentially jeopardize a successful breastfeeding. However, the demand for DM is a reality for the infants at the NICUs, and women need to be able to make their own informed decisions based on information that should be made available to them.

The researchers interviewing these women both have extensive experience from neonatal intensive care and are aware of the importance of breast milk donors. This might have had the potential to introduce reflexivity bias. By being conscious and acknowledging that we reflected on this throughout the project and adopted an open-minded stance during the analysis reducing the risk of bias effecting the results.

### Limitations

These results are based on a relatively small sample of breast milk donors in Sweden and might not be generalizable to all women.

## Conclusions

Despite the challenges posed by COVID-19 restrictions, time consumption, and the hard work of sterilizing pump utensils, women continued to donate their milk driven by altruism. To enhance donor support and increase milk donation, several improvements are suggested: providing comprehensive information and resources, simplifying the donation process, offering flexible scheduling, and recognizing donors’ contributions.

### Electronic supplementary material

Below is the link to the electronic supplementary material.

Supplementary Material 1

## Data Availability

The data presented in this study are available on request from the corresponding author. The data are not publicly available due to the statutory requirements, internal data policies, and regulations existing in the collaborating bodies along with the over-arching General Data Protection Regulation (GDPR) stating that the data must be stored in an institutional repository (storage platform).

## Abbreviations

COVID-19: Coronavirus disease 2019
DM: Donor milk
MOM: Mother’s own milk
NEC: Necrotizing enterocolitis
NICU: Neonatal intensive care unit
