# Supplementary material for: Experiences of breast milk donors in Sweden: balancing the motivation to do something good with overcoming the challenges it entails
Source: Int Breastfeed J. 2024 Aug 31;19:60. doi: 10.1186/s13006-024-00668-3 (PMC11365258; doi:10.1186/s13006-024-00668-3)
Supplement: Supplementary file 1 — Supplementary Material 1 [file 13006_2024_668_MOESM1_ESM.docx]

Additional file 1.

**Interview guide**

Initial question: Please tell me about your experiences of being a breast milk donor.

- What made you want to donate breast milk
  - Where/how did you get information about the possibility to donate?
- How were you met by the milk bank before, during and after the donation period?
- How did you execute the donation practically;
  - Did you also breastfeed your infant?
  - When did you pump out the milk?
  - How did the hospital receive your milk?
  - How long did it take you to donate?
- Were there any facilitating factors?
- Were there any hindering factors?
- Could the staff at the milk bank had done something else to support you during the donation period?
- Do yo have any advice to the staff at the milk bank that you would like to share?
- What made you stop donating breast milk?
- Is there anything else you would like to tell me regarding your experience of donating breast milk?
